# Comparative short-term efficacy of Janus kinase 1 inhibitors and anti-interleukin-13 antibodies in atopic dermatitis: a retrospective cohort analysis based on real-world data

**DOI:** 10.3389/fimmu.2025.1639932

**Published:** 2025-08-11

**Authors:** Emi Sato, Naoko Obonai, Mayuko Iwata, Kotaro Ito, Shinichi Imafuku

**Affiliations:** ^1^ Department of Dermatology, Faculty of Medicine, Fukuoka University, Fukuoka, Japan; ^2^ Department of Dermatology, Ito Dermatology Clinic, Kitsuki, Oita, Japan

**Keywords:** Janus kinase 1 inhibitors, anti-IL-13 antibodies, IL-31, pruritus, IL-5, eosinophils, TARC, atopic dermatitis

## Abstract

**Introduction:**

Molecular targeted therapies, including advanced atopic dermatitis (AD) treatment with Janus kinase 1 inhibitors (JAK1i) and anti-interleukin-13 antibodies (IL-13Ab), are emerging as effective options. However, the predictive biomarkers for treatment responses remain unclear. Therefore, this study compared the short-term efficacy of JAK1i and IL-13Ab and explored relevant biomarkers.

**Methods:**

This retrospective analysis was conducted in 75 patients with moderate-to-severe AD treated at Fukuoka University Hospital. Relevant biomarkers, including eosinophil count and thymus and activation-regulated chemokine (TARC) levels, were measured at baseline and 3 months. Eczema Area and Severity Index (EASI) and Peak Pruritus Numerical Rating Scale (PP-NRS) scores were also assessed.

**Results:**

Patients received JAK1i (n=37; abrocitinib, n=16; upadacitinib, n=21) or IL-13Ab (n=38; lebrikizumab, n=21; tralokinumab, n=17). At 3 months, no significant difference was observed between JAK1i and IL-13Ab in achieving EASI 75 (odds ratio [OR] = 0.83, p=0.76) or in the incidence of adverse events (OR = 1.40, p=0.55). However, JAK1i was associated with higher odds of achieving PP-NRS 4 (OR=9.36, p=0.0063) and PP-NRS 0/1 (OR=34.61, p<0.0001). In the JAK1i group, eosinophil count reduction correlated with EASI improvement (univariate: R=0.525, p=0.0009; adjusted: β = 0.567, p=0.0004). In the IL-13Ab group, TARC reduction correlated with EASI improvement (univariate: R=0.677, p<0.0001; adjusted: β = 0.661, p<0.0001).

**Conclusion:**

JAK1i showed greater antipruritic effects than IL-13Ab at 3 months, likely due to interleukin (IL)-31 inhibition. Eosinophil count reduction was the most reflective biomarker of JAK1i efficacy, potentially due to IL-5 suppression, whereas TARC improvement was significantly associated with patients’ treatment response to IL-13Ab. These findings highlight the need for further long-term studies.

## 1 Introduction

Atopic dermatitis (AD) is a chronic inflammatory skin disease characterized by Th2-dominant immune dysregulation (1–3). In recent years, several molecular-targeted therapies, including Janus kinase (JAK) inhibitors and biologics targeting interleukin (IL)-13, have been introduced into clinical practice (4, 5). Selecting the appropriate therapy requires consideration of not only efficacy and safety, but also patient background characteristics and predictive biomarkers for treatment response.

Selective JAK1 inhibitors (JAK1i) suppress multiple inflammatory pathways, including IL-4/13, IL-31, and TSLP, thus offering broad clinical benefits (6–8). Conversely, anti-IL-13 antibodies (IL-13Ab), e.g., tralokinumab and lebrikizumab, selectively block the IL-13 pathway and modulate inflammation and skin barrier function (9–11).

The predictive biomarkers for treatment responses remain unclear. Therefore, this study aimed to retrospectively compare the clinical outcomes and safety of JAK1i and IL-13Ab and to identify clinical characteristics and biomarkers associated with treatment response or adverse events to support individualized therapeutic strategies. To clarify the cytokine pathway-specific effects, we selected agents that target IL-13 alone and selective JAK1 inhibitors. The IL-4/13 receptor blockers, such as dupilumab, were excluded because they concurrently inhibit both IL-4 and IL-13 signaling, making it difficult to isolate the specific contribution of IL-13 inhibition on clinical outcomes and biomarker changes. Similarly, JAK1/2 inhibitors such as baricitinib were excluded to allow precise evaluation of JAK1-selective inhibition.

## 2 Materials and methods

### 2.1 Study design

This retrospective cohort study included patients with moderate-to-severe AD treated with either JAK1i (abrocitinib or upadacitinib) or IL-13Ab (tralokinumab or lebrikizumab) at the Department of Dermatology, Fukuoka University Hospital between January 2021 and March 2024. AD was diagnosed according to the criteria established by the Japanese Dermatological Association (12). Patients enrolled in clinical trials or with missing data for any of the variables required for analysis (baseline and 3-month EASI, PP-NRS, eosinophil count, serum IgE, and TARC) were excluded. Propensity score matching or sensitivity analyses were not conducted due to the limited sample size; however, multivariate logistic regression was used to adjust for potential confounding variables.

### 2.2 Analysis of patients treated with JAK1i or anti-IL-13Ab

The following baseline characteristics were evaluated: age, sex, bio-naïve status, history of systemic therapy, baseline Eczema Area and Severity Index (EASI), Peak Pruritus Numerical Rating Scale (PP-NRS) score, serum levels of TARC and total immunoglobulin (Ig)-E, and peripheral blood eosinophil count. Additionally, EASI and PP-NRS scores were reassessed at 3 months, and the achievement of EASI 75 (≥75% improvement), PP-NRS 4 (≥4-point improvement), and PP-NRS 0/1 (score of 0 or 1) were defined as primary clinical outcomes. Adverse events were defined as treatment-related events recorded during the treatment period. The improvement rates of the biomarkers (TARC, IgE, and eosinophils) were calculated on the basis of the change from baseline to 3 months.

### 2.3 Statistical analysis

Group comparisons of patient characteristics were performed using the Mann–Whitney U test or Fisher exact test. Logistic regression analysis adjusted for common confounding variables (treatment group, age, sex, bio-naïve status, history of systemic therapy, and baseline scores) was performed to identify the factors associated with each clinical outcome. Additionally, simple linear regression analyses were performed to assess the associations between EASI or PP-NRS improvement rates and biomarker improvement rates, and correlation coefficients (R) and corresponding p-values were reported. For multiple linear regression models, the coefficient of determination (R²) and adjusted R² were used to evaluate model performance. All statistical analyses were performed using GraphPad Prism (version 5; GraphPad Software, Inc.) and JMP (version 14.2.0; SAS Institute). Statistical significance was set at p < 0.05.

### 2.4 Ethics statement

This study was approved by Fukuoka University - Medical Ethics Review Board (approval number: U21-08-004). Some of the enrolled patients overlapped with those in previous reports; however, this study involved a new comparative analysis of the treatment groups and their associated biomarkers. The study period and protocol were updated in June 2024 and approved by an additional application (approval number: U24-947). This study was conducted in accordance with the Declaration of Helsinki and the Japanese Ethical Guidelines for Medical Research Involving Human Subjects. Information regarding the study objectives and methodology was disclosed on the hospital website, and informed consent was obtained through an opt-out process. None of the patients declined participation.

## 3 Results

### 3.1 Patient demographic characteristics

As shown in **Table 1**, the median ages of patients were 38 years in the JAK1i group and 57 years in the IL-13Ab group, with a significant difference between the groups. The median baseline EASI score was significantly higher in the JAK1i group than in the IL-13Ab group (26.5 versus [*vs*.] 18.7). The JAK1i group also showed significantly higher baseline IgE (4071 *vs*. 1585 IU/mL) and TARC levels (2949 *vs*. 1901 pg/mL).

**Table 1:** Baseline characteristics of patients using JAK1 inhibitors or anti-IL-13 antibodies.

Baseline	JAK1 inhibitor		IL-13 antibody		p-value
**Total, N**	**37**		**38**		
**Women, N (%)**§	12	(31.58)	16	(42.11)	0.476
**Asian, N (%)**§	37	(100.00)	38	(100.00)	1.0
**Age, y, median (Q1/Q3)** ¶	38	(23/50)	57	(37/73.5)	0.0054*
**Atopic predisposition, N (%)**§	36	(97.30)	33	(86.84)	0.1997
**EASI score, median (Q1/Q3)** ¶	26.5	(18.7/38.45)	18.7	(16.475/24.225)	0.0019*
**PP-NRS score, median (Q1/Q3)** ¶	7	(4.5/8)	6	(3/9)	0.4265
**Bio-naïve, N (%)**§	26	(70.27)	20	(52.63)	0.156
**Systemic therapy-naïve, N (%)**§	12	(32.43)	11	(28.95)	0.8054
**Lebrikizumab, total, N (%)**	-	-	**21**	(55.26)	
**Tralokinumab, total, N (%)**	-	-	**17**	(44.74)	
**Abrocitinib, total, N (%)**	**16**	(43.24)	-	-	
100mg, N (%)	12	(32.43)	-	-	
200mg, N (%)	4	(10.81)	-	-	
**Upadacitinib, total, N (%)**	**21**	(56.76)	-	-	
15mg, N (%)	13	(35.14)	-	-	
30mg, N (%)	8	(21.62)	-	-	
**Eosinophil count, median (Q1/Q3)** ¶	455	(243/775.25)	416.4	(249.35/728.25)	0.7667
**Serum IgE, median (Q1/Q3)** ¶	4071	(1735.5/12166)	1585	(298.75/5628.25)	0.0271*
**Serum TARC, median (Q1/Q3)** ¶	2949	(1185.5/7021)	1901	(457.5/3445)	0.0142*

§ Fisher exact test, ¶ Mann–Whitney test, *Statistically significant at p < 0.05. JAK1, Janus kinase 1; IL-13, interleukin-13; Q1, quartile 1; Q3, quartile 3; EASI, Eczema Area and Severity Index; PP-NRS, Peak Pruritus Numerical Rating Scale; IgE, immunoglobulin E; TARC, thymus and activation-regulated chemokine.

Bold values* indicate statistically significant differences (p < 0.05).

No significant differences were observed between the groups in terms of sex, atopic predisposition, baseline PP-NRS score, bio-naïve status, history of systemic therapy, or eosinophil count. Patients received lebrikizumab or tralokinumab in the IL-13Ab group, and abrocitinib or upadacitinib in the JAK1i group.

### 3.2 Univariate and multivariate analysis of treatment outcomes

**Figure 1:**
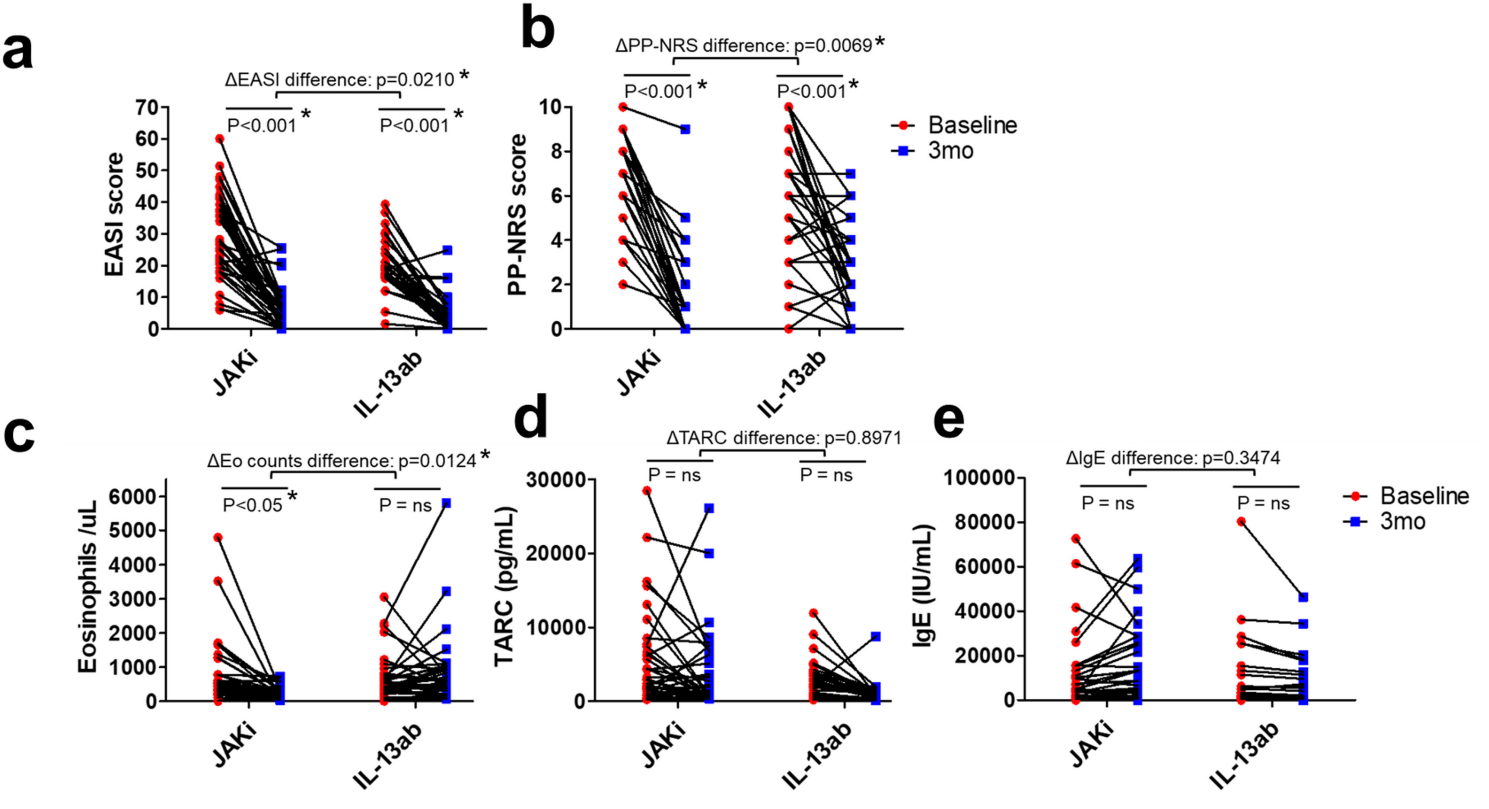
Individual patient changes in clinical scores and biomarkers from baseline to 3 months in the JAK1i and IL-13Ab groups. **(a)** EASI scores, **(b)** PP-NRS scores, **(c)** peripheral blood eosinophil counts, **(d)** serum TARC levels, and **(e)** total serum IgE levels are shown. Each line represents an individual patient’s data. Statistical comparisons were performed using two-way ANOVA with Bonferroni post-tests. * p < 0.05. JAK1i, Janus kinase 1 inhibitors; IL-13Ab, interleukin-13 antibodies; EASI, Eczema Area and Severity Index; PP-NRS, Peak Pruritus Numerical Rating Scale; IgE, immunoglobulin E; ANOVA, analysis of variance; TARC, thymus and activation-regulated chemokine.

As shown in **Table 2** and **Figure 1b**, the JAK1i group showed significantly greater improvements in PP-NRS scores, PP-NRS improvement rates, PP-NRS 4 achievement, and PP-NRS 0/1 achievement than the IL-13Ab group. However, no significant differences were observed between the two groups in terms of EASI scores, EASI improvement rates, or EASI 75 achievement (**Table 2**). However, two-way analysis of variance (ANOVA) for EASI improvement (ΔEASI) revealed a significant difference between the treatment groups, with the JAK1i group showing greater improvement than the IL-13Ab group (**Figure 1a**).

**Table 2:** Results following JAK1 inhibitor or anti-IL-13 antibody treatment.

(Three months after treatment)	JAK1 inhibitor		IL-13 antibody		p-value
**Total, N**	**37**		**38**		
**PP-NRS score, median (Q1/Q3)** ¶	0	(0/2.5)	2	(2/4)	0.0003*
PP-NRS improvement rate, %, median (Q1/Q3) ¶	100	(59.82/100)	60	(0/73.02)	< 0.0001*
PP-NRS 4 achievement, N (%)§	30	(81.08)	17	(44.74)	0.0017*
PP-NRS 0/1 achievement, N (%)§	27	(72.97)	8	(21.05)	< 0.0001*
**EASI score, median (Q1/Q3)** ¶	4.1	(0/8.1)	3.45	(1.2/5.93)	0.6734
EASI improvement rate, %, median (Q1/Q3) ¶	83.33	(74.41/100)	79.98	(65.8/94.16)	0.4280
EASI 75 achievement, N (%)§	27	(72.22)	27	(71.05)	1.0
**Eosinophil count, median (Q1/Q3)** ¶	263	(124.5/356.5)	557	(206/951)	< 0.0001*
Eosinophil change rate, %, median (Q1/Q3) ¶	56.83	(11.59/82.67)	-25.92	(-150.4/24.88)	< 0.0001*
**Serum IgE, median (Q1/Q3)** ¶	5354	(1401.5/23289.5)	1014	(239.75/6932.75)	0.0051*
IgE change rate, %, median (Q1/Q3) ¶	7.96	(-20.54/34.45)	21.48	(4.73/35.51)	0.2591
**Serum TARC, median (Q1/Q3)** ¶	1384	(596/5711.5)	531	(370.5/1061.75)	0.0005*
TARC change rate, %, median (Q1/Q3) ¶	37.22	(-46.71/80.71)	55.01	(17.52/79.4)	0.1667
**Patient with adverse events, N (%)**§	17	(45.95)	15	(39.47)	0.6438
Total adverse events, N	26		18		

§ Fisher exact test, ¶ Mann–Whitney test, *Statistically significant at p < 0.05. JAK1, Janus kinase 1; IL-13, interleukin-13; Q1, quartile 1; Q3, quartile 3; EASI, Eczema Area and Severity Index; PP-NRS, Peak Pruritus Numerical Rating Scale; IgE, immunoglobulin E; TARC, thymus and activation-regulated chemokine.

Bold values* indicate statistically significant differences (p < 0.05).

Regarding biomarkers, the eosinophil count decreased significantly in the JAK1i group, whereas little change was observed in the IL-13Ab group; the between-group difference in change rates was also significant (**Table 2**, **Figure 1c**). There were no significant differences in the TARC and IgE change rates between the groups (**Table 2**, **Figure 1d, e**). However, a significant reduction in TARC levels was observed in the IL-13Ab group after treatment (p=0.0016 [paired t-test]; data not shown).

As shown in **Table 3**, multivariate logistic regression analysis adjusted for common confounding factors identified JAK1i as significant independent predictors for achieving PP-NRS 4 (odds ratio [OR]=9.36) and PP-NRS 0/1 (OR=34.61). Conversely, no significant predictors were identified for EASI 75 or adverse events.

**Table 3:** Comparison of multivariable logistic regression models for the four outcomes.

Variable	EASI75 (OR) [95% CI], p	PP-NRS4 (OR) [95% CI], p	PP-NRS 0/1 (OR) [95% CI], p	Adverse event (OR) [95% CI], p
**JAKi (*vs* IL-13Ab)**	0.83 [0.25–2.79], 0.76	**9.36** [1.88–46.52], 0.0063*	**34.61** [5.93–202.04], <0.0001*	1.4 [0.47–4.18], 0.55
**Female (*vs* Male)**	2.7 [0.74–9.90], 0.13	0.81 [0.24–6.28], 0.80	1.1 [0.25–3.29], 0.88	1.68 [0.20–1.73], 0.34
**Age**	1.01 [0.98–1.04], 0.61	2.55 [0.27–24.69], 0.61	1.01 [0.98–1.04], 0.51	0.99 [0.96–1.01], 0.69
**Bio-naïve**	1.37 [0.42–4.39], 0.60	1.62 [0.36–7.29], 0.53	1.23 [0.33–4.61], 0.76	0.62 [0.21–1.78], 0.37
**Systemic therapy-naive**	0.71 [0.20–2.51], 0.60	0.44 [0.07–2.64], 0.37	1.33 [0.38–4.75], 0.66	0.42 [0.14–1.28], 0.13
**Baseline EASI**	1.07 [0.99–1.16], 0.69	52.48 [0.91–7376.45], 0.69	0.98 [0.91–1.02], 0.34	0.99 [0.93–1.04], 0.66
**Baseline PP-NRS**	1.07 [0.82–1.40], 0.59	**2.06** [1.47–29.40], 0.0012*	**0.67** [0.48–0.89], 0.0088*	1.09 [0.86–1.41], 0.46

Model summary: Each column represents a separate multivariable logistic regression model adjusted for the listed covariates. Pseudo-R² ranged from 0.06–0.46, and the global model fit was not statistically significant in some analyses (EASI75 and adverse event).

Odds ratios (ORs), 95% confidence intervals (CIs), and p-values are shown for each outcome. To allow direct comparison across outcomes, a uniform set of covariates was used for all models. These uniform models may not represent the optimal covariate set for each outcome. The outcome-specific models with variable selection and improved fit are provided in **Supplementary Table S1**.

* Statistically significant at p < 0.05.

JAK1, Janus kinase 1; IL-13, interleukin-13; EASI, Eczema Area and Severity Index; PP-NRS, Peak Pruritus Numerical Rating Scale; IgE, immunoglobulin E; *vs*, versus; EASI75, Eczema Area and Severity Index of 75; PP-NRS4, Peak Pruritus Numerical Rating Scale score of 4; PP-NRS 0/1, Peak Pruritus Numerical Rating Scale score of 0 or 1.

Bold values* indicate statistically significant differences (p < 0.05).

In the optimized supplementary model (**Supplementary Table S1**), the baseline EASI score, TARC improvement rate, and IgE improvement rate were significant predictors of EASI 75 achievement. The baseline IgE level and TARC improvement rate were associated factors with adverse events, although their ORs were close to 1, indicating a limited predictive effect.

### 3.3 Biomarker analysis

**Figure 2:**
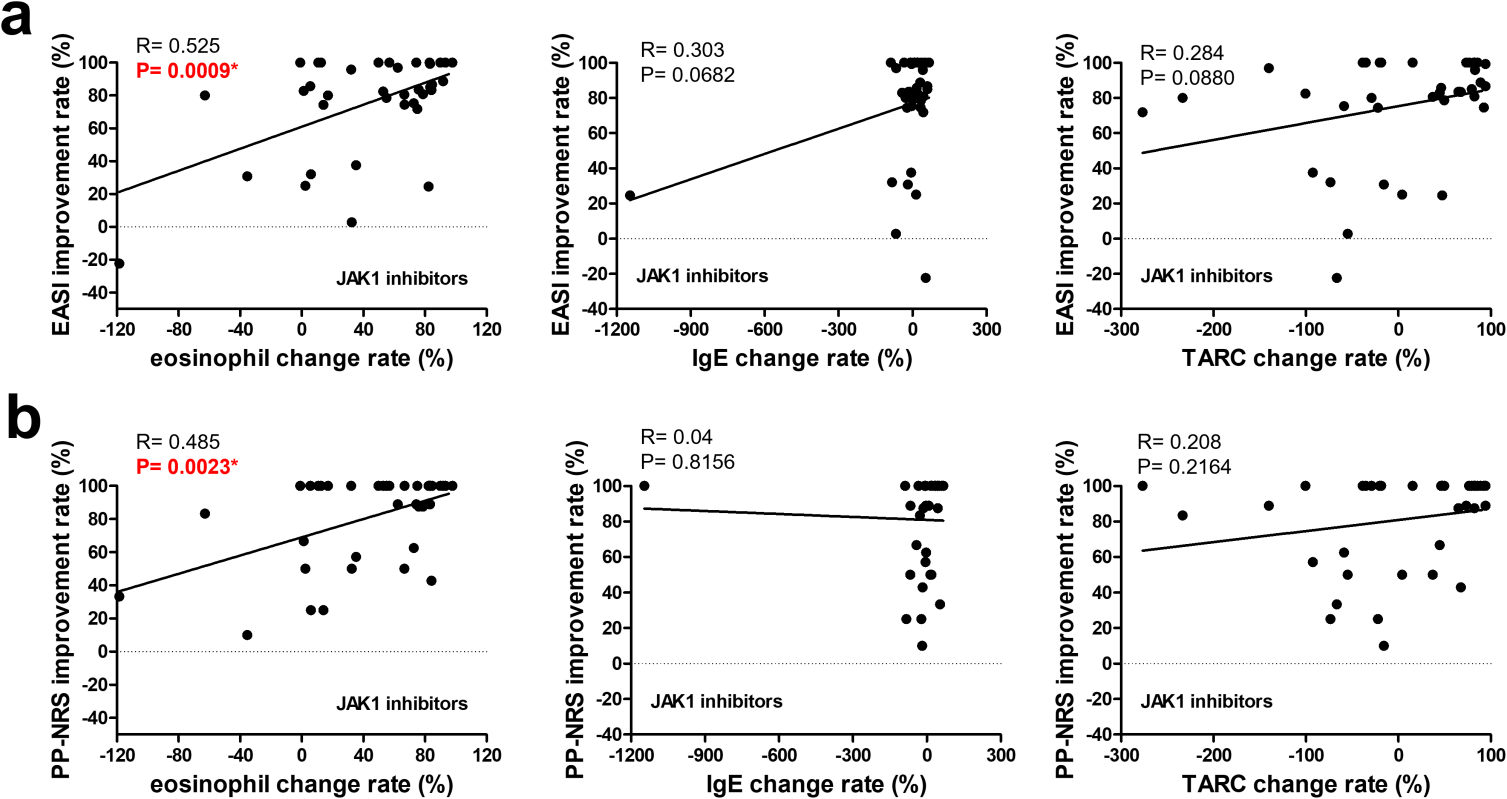
Correlation between changes in biomarkers and improvement rates in clinical outcomes at 3 months in patients treated with JAK1 inhibitors. The upper row **(a)** shows the correlations between the improvement rates in the EASI and changes in (left to right) the eosinophil counts, IgE levels, and TARC levels. The lower row **(b)** shows the correlations between the improvement rates in the PP-NRS and changes in (left to right) eosinophil counts, IgE levels, and TARC levels. A simple linear regression analysis was used for all correlations. R and p-values are indicated. * p < 0.05. EASI, Eczema Area and Severity Index; PP-NRS, Peak Pruritus Numerical Rating Scale; IgE, immunoglobulin E; TARC, thymus and activation-regulated chemokine.

**Figure 3:**
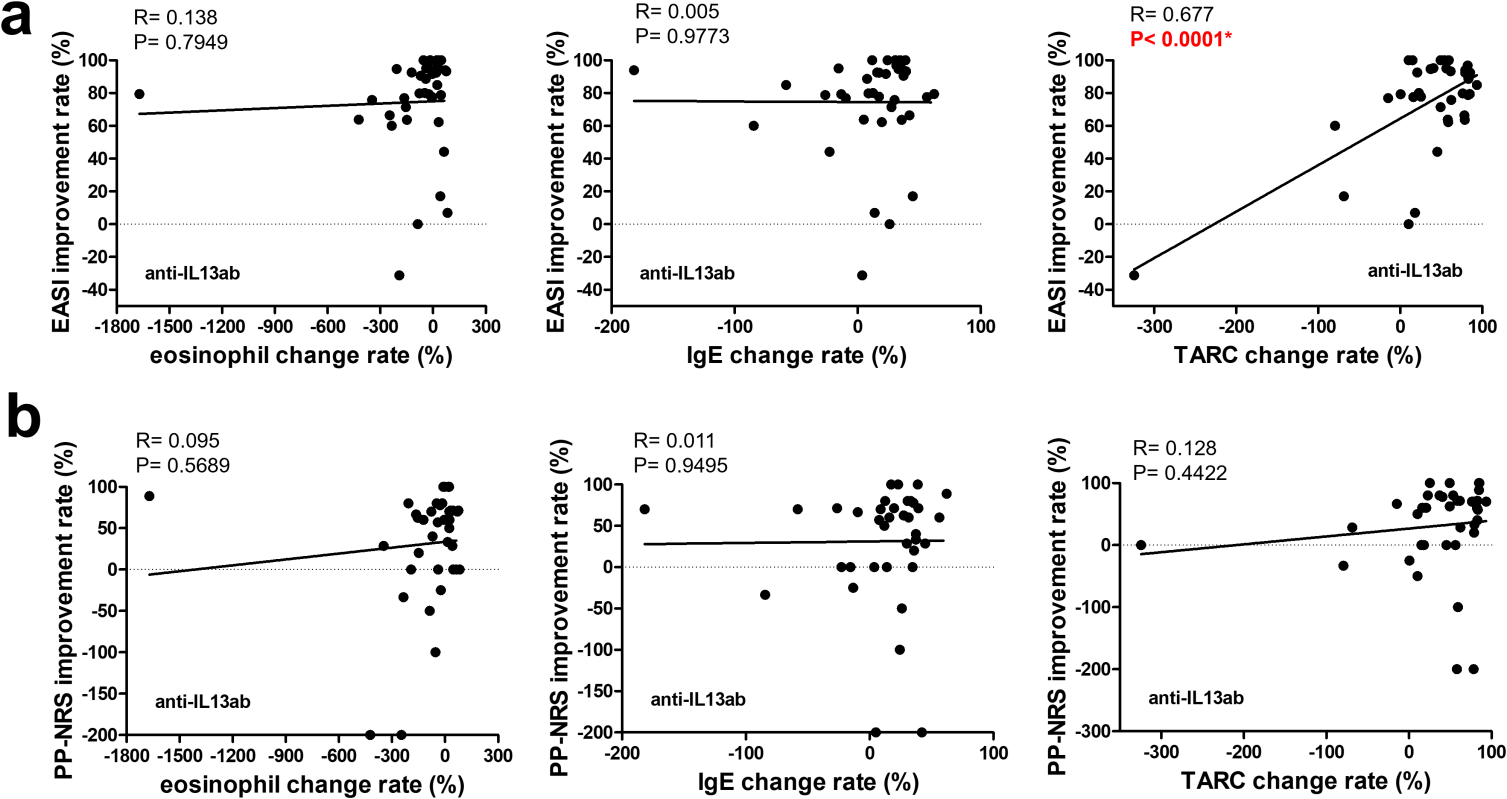
Correlation between changes in biomarkers and improvement rates in clinical outcomes at 3 months in patients treated with anti-IL-13 antibodies. The upper row **(a)** shows the correlations between the improvement rates in the EASI and changes in (left to right) the eosinophil counts, IgE levels, and TARC levels. The lower row **(b)** shows the correlations between the improvement rates in the PP-NRS and changes in (left to right) the eosinophil counts, IgE levels, and TARC levels. A simple linear regression analysis was used for all correlations. R and p-values are indicated. * p < 0.05. EASI, Eczema Area and Severity Index; PP-NRS, Peak Pruritus Numerical Rating Scale; IgE, immunoglobulin E; TARC, thymus and activation-regulated chemokine.

As shown in **Figure 2**, the eosinophil improvement rate in the JAK1i group significantly correlated with EASI improvement and PP-NRS improvement, according to simple linear regression analysis. However, in the IL-13 antibody group (**Figure 3**), only the TARC improvement rate was significantly correlated with EASI improvement.

According to multiple linear regression analyses shown in **Table 4**, the biomarkers significantly associated with EASI improvement in the JAK1i group were eosinophil improvement rate, consistent with the simple regression result, whereas no significant associations were observed with PP-NRS improvement (**Supplementary Table S2a**). Similarly, in the IL-13 antibody group, the TARC improvement rate remained significantly associated with EASI improvement (**Table 5**). Although no statistically significant biomarker associations were identified with PP-NRS improvement (**Supplementary Table S2b**), a significant negative association was found between baseline IgE levels and improvement in the IL-13Ab group (**Supplementary Table S2b**).

**Table 4:** Biomarkers correlated with 3-month EASI improvement in patients treated with JAK inhibitors.

Biomarker correlated with EASI Improvement	Standardized β	Raw β [95% CI]	*p*-value	VIF
**Eosinophil Improvement Rate**	**0.567**	**0.362 [0.175, 0.548]**	0.0004*	**1.26**
**IgE Improvement Rate**	**0.345**	**0.055 [0.012, 0.097]**	0.0141*	**1.08**
**TARC Improvement Rate**	0.097	0.033 [–0.079, 0.144]	0.5548	1.62

Model Summary: N = 37, R² = 0.512 (adjusted R² = 0.415), RMSE = 23.37, model (p) = 0.0008, no multicollinearity (VIF < 2 for all variables).

* Statistically significant at p < 0.05.

The full multivariable model also included age, history of systemic treatment, and history of biologic use, none of which were statistically significant. Only biomarker-related variables are presented in the table for clarity.

CI, confidence interval; EASI, Eczema Area and Severity Index; IgE, immunoglobulin E; TARC, thymus and activation-regulated chemokine; VIF, variance inflation factor; RMSE, root mean square error.

Bold values* indicate statistically significant differences (p < 0.05).

**Table 5:** Biomarkers correlated with 3-month EASI improvement in patients treated with anti-IL-13 antibodies.

Biomarker correlated with EASI Improvement	Standardized β	Raw β [95% CI]	*p*-value	VIF
**TARC Improvement Rate**	**0.661**	**0.277 [0.156, 0.397]**	<0.0001*	**1.35**
**IgE Improvement Rate**	–0.034	–0.024 [–0.209, 0.162	0.7963	1.15
**Eosinophil Improvement Rate**	0.013	0.001 [–0.026, 0.028]	0.9171	1.07

Model Summary: N = 38, R² = 0.542 (Adjusted R² = 0.453), RMSE = 22.38, model (p) = 0.0003, No multicollinearity (VIF < 2 for all variables).

* Statistically significant at p < 0.05.

The full model also included baseline EASI, baseline IgE, and bio-naïve status, none of which were statistically significant. Only biomarker-related variables are presented in the table for clarity.

CI, confidence interval; EASI, Eczema Area and Severity Index; IgE, immunoglobulin E; TARC, thymus and activation-regulated chemokine; VIF, variance inflation factor; RMSE, root mean square error.

Bold values* indicate statistically significant differences (p < 0.05).

### 3.4 Comparison of adverse events

As shown in **Tables 2**, **6**, the incidences of adverse events were 45.95% in the JAK1i group and 39.47% in the IL-13 antibody group, with no significant difference between the groups (**Table 2**). In the JAK1i group, the most common adverse events were acne or folliculitis, viral warts, and herpes zoster. Temporary discontinuation or treatment cessation was required in 5 of the 17 cases. In the IL-13 antibody group, ocular pruritus and conjunctivitis were the most frequently reported symptoms, followed by single reports of fatigue, asthma exacerbation, and grade 3 eosinophilia. Treatment discontinuation due to adverse events was required in three cases in the IL-13 antibody group. Severe adverse events were rare in both groups (**Table 6**).

**Table 6:** Summary of adverse events in the JAK1 inhibitor and anti-IL-13 antibody groups.

a) JAK1 inhibitors.	
JAK1 inhibitor(N=37)	N
**Number of patient with adverse events**	**17**
**(Number of total adverse events)**	**(26)**
Acne, folliculitis	9
*^$^Viral warts	4
*Herpes zoster	3
Dyslipidemia	2
Eczema herpeticum	1
*Liver dysfunction	1
Impetigo	1
*Erysipelas	1
*Neutropenia, grade 3	1
Nausea	1
Headache	1
^+^Toxic eruption	1
b) Anti-IL-13 antibodies.	
Anti-IL-13 antibody(N=38)	N
**Number of patient with adverse events**	**15**
**(Number of total adverse events)**	**(18)**
Ocular pruritus/conjunctivitis	10
Cutaneous pruritus/prurigo nodularis	2
Arthralgia	1
*Eosinophilia, grade 3	1
^+^Granulomatous dermatitis	1
^+^Fatigue	1
*Exacerbation of asthma	1
*^+^Purpuric drug eruption	1

* Adverse events leading to temporary or permanent discontinuation.

$ Viral warts: condyloma acuminatum (1 patient), molluscum contagiosum (1 patient), verruca plana (2 patients).

+ Causality unclear.

JAK1, Janus kinase 1; IL-13, interleukin-13.

Bold values* indicate statistically significant differences (p < 0.05).

## 4 Discussion

This retrospective cohort study examined the short-term efficacy and biomarker profiles of selective JAK1i and IL-13Ab in moderate-to-severe AD. Despite comparable skin clearance rates (EASI 75), JAK1i exhibited more pronounced antipruritic effects. Although these antipruritic benefits may partly reflect baseline differences or prescribing patterns in clinical practice, the consistent and greater improvement in pruritus observed with JAK1i suggests a potentially more effect on itch pathways, particularly those involving IL-31—a key mediator of pruritus in AD not targeted by IL-13Ab (13–15) —as well as its broader inhibition of key Th2-related cytokines such as IL-4 and TSLP (6, 16–18), resulting in enhanced antipruritic efficacy. These findings are further supported by a recent real-world study by Ibba et al., which demonstrated marked improvement in pruritus and disease severity with both abrocitinib and upadacitinib during the early phase of treatment, reinforcing the short-term antipruritic efficacy of JAK1i in clinical practice (19). In our cohort, distinct biomarker associations were also observed, suggesting differences in the underlying mechanisms of action between JAK1i and IL-13Ab. However, given the retrospective design of our study, potential selection bias and residual confounding cannot be ruled out, and these findings should be interpreted with appropriate caution.

Interestingly, while the overall EASI 75 achievement rates were comparable between the groups, biomarker analyses revealed pathway-specific correlates of treatment response. In the JAK1i group, reductions in eosinophil counts were strongly associated with EASI improvement, suggesting that eosinophils may be associated with treatment response. This is consistent with the ability of JAK1 inhibition to suppress IL-5 signaling and downstream eosinophilic inflammation. Supporting this, Song et al. reported that abrocitinib significantly reduced peripheral eosinophil counts in patients with moderate-to-severe AD, and that this reduction correlated with clinical improvement (20). Similarly, Hagino et al. demonstrated that total eosinophil count (TEC) reductions were significantly associated with both EASI and PP-NRS improvements during 48 weeks of upadacitinib treatment, reinforcing the potential utility of eosinophils as long-term biomarkers in JAK1i therapy (21). Furthermore, in their recent publication (22), longitudinal data up to 96 weeks clearly illustrated parallel declines in eosinophil counts and EASI scores, particularly in the bio-naive group, further supporting the sustained clinical relevance of TEC dynamics throughout extended JAK1i treatment.

In contrast, in the IL-13Ab group, TARC (CCL17) reduction was most significantly correlated with EASI improvement. TARC is a key Th2 chemokine produced by keratinocytes and dendritic cells in response to IL-13 stimulation via JAK1/STAT6 signaling (23, 24). By directly neutralizing IL-13, tralokinumab and lebrikizumab suppress TARC gene transcription and reduce Th2 cell recruitment to the skin, contributing to inflammation resolution (25, 26). This mechanism is distinct from that of JAK1i, which broadly inhibits multiple cytokine pathways—including IL-4, IL-5, and IL-31—whereas IL-13Abs specifically target IL-13–mediated responses such as TARC production. Therefore, TARC reduction may serve as a surrogate biomarker of IL-13 pathway inhibition and clinical efficacy in patients treated with IL-13Abs. Notably, a significant negative association was found between baseline IgE levels and pruritus improvement in the IL-13 antibody group, warranting further investigation.

Since IL-13 antibodies and IL-31 receptor blockade do not inhibit IL-4 signaling, they are less effective in suppressing serum IgE levels. This may explain why patients with higher baseline IgE levels tended to show less improvement in pruritus with these treatments. In support of this notion, a recent study of nemolizumab, an anti-IL-31 receptor antibody, reported that lower baseline IgE levels were significantly associated with PP-NRS 4 achievement (27). These findings suggest that IgE may influence the pruritus response, particularly in response to therapies that do not block the IL-4 pathway. Although not shown here, we confirmed in a separate analysis that serum IgE levels significantly decreased at 3 months in patients treated with IL-4/13 receptor antibodies (dupilumab), whereas no significant reduction was observed in those treated with IL-13 antibodies alone (tralokinumab or lebrikizumab). This further supports the concept that the suppression of IL-4 signaling is necessary for effective IgE reduction and may contribute to enhanced antipruritic effects in patients with elevated baseline IgE levels. On the other hand, Hagino et al. separately evaluated 126 patients treated with lebrikizumab (28) and 106 with tralokinumab (29), and reported that both TARC and IgE levels significantly decreased—at week 16 in the lebrikizumab group and at week 24 in the tralokinumab group. These findings suggest that when using IL-13–targeted therapies, a longer observation period may be required to assess serum IgE responses compared to IL-4/13 receptor blockade.

Importantly, our study is the first to directly compare selective JAK1i and IL-13Ab, with a focus on pathway-specific biomarkers. While previous studies, such as those by Song et al. and Hagino et al., have investigated biomarker trajectories following treatment with either JAK1 inhibitors or IL-13 antibodies, our retrospective study is the first to offer a direct comparison between these two therapeutic classes and to show that reductions in eosinophils and TARC are differentially associated with clinical response, depending on the targeted cytokine pathway. This direct comparison provides novel insights into personalized therapeutic strategies for AD.

Both treatment modalities were well tolerated, with no significant differences in the incidence of adverse events. However, adverse event profiles differed according to the mechanism of action. The JAK1i group exhibited higher rates of acne, folliculitis, viral warts, and herpes zoster than the IL-13Ab group, reflecting the broader immunosuppressive effects of JAK1 inhibition, including impaired type I and II interferon signaling crucial for antiviral defense (30). Pre-existing viral warts, including flat warts, molluscum contagiosum, and condyloma acuminatum, may be masked by eczematous inflammation in AD and become clinically apparent only after resolution of skin lesions with JAK1i therapy (31). Therefore, regular screening for latent viral infections is advisable during JAK1i therapy. Conversely, conjunctivitis and ocular pruritus were more common in the IL-13Ab group, likely due to the role of IL-13 in maintaining conjunctival goblet cell homeostasis and tear film stability; its blockade may promote ocular surface inflammation and dry eye symptoms (32–34). Although the types of adverse events differed between groups, both treatments were generally well tolerated, with few severe events or discontinuations. This is consistent with an umbrella review by He et al., which found that JAK inhibitors like abrocitinib and upadacitinib are effective but more often cause acne and gastrointestinal symptoms (35).

This study has some limitations. Its retrospective design, modest sample size, and relatively short 3-month follow-up limit the generalizability and preclude evaluation of long-term outcomes—an important consideration in chronic diseases like AD. Additionally, baseline imbalances in age, EASI scores, and serum biomarkers such as TARC and IgE between the JAK1i and IL-13Ab groups may have introduced residual confounding that could not be fully eliminated by multivariate adjustment. These imbalances reflect differences in prescribing patterns in clinical practice, and may have influenced both clinical outcomes and biomarker responses. Therefore, caution is warranted in interpreting direct comparisons between the two groups, and prospective randomized studies are needed to confirm these findings.

In conclusion, selective JAK1i demonstrated greater short-term antipruritic effects than IL-13Ab, and treatment responses were associated with different biomarker profiles. These results underscore the importance of cytokine-targeted selection in clinical practice and suggest that eosinophil and TARC reductions are associated with treatment responses, potentially aiding individualized AD treatments. Further prospective studies with larger cohorts and longer follow-up periods are warranted to validate these findings and to establish long-term treatment algorithms.

## Data Availability

The raw data supporting the conclusions of this article will be made available by the authors, without undue reservation.
