# Supplementary material for: Comparative short-term efficacy of Janus kinase 1 inhibitors and anti-interleukin-13 antibodies in atopic dermatitis: a retrospective cohort analysis based on real-world data
Source: Front Immunol. 2025 Aug 11;16:1639932. doi: 10.3389/fimmu.2025.1639932 (PMC12375639; doi:10.3389/fimmu.2025.1639932)
Supplement: Supplementary file 1 [file Table1.docx]

Supplementary Data

# Supplementary Table S1

| **Variable** | **EASI75 (OR [95% CI], p)** | **Adverse Event (OR [95% CI], p)** |
| --- | --- | --- |
| **Treatment (JAKi vs IL-13Ab)** | 0.53 [0.12–2.14], 0.38 | 0.70 [0.19–2.40], 0.57 |
| **Age** | 1.01 [0.98–1.04], 0.44 | 0.99 [0.97–1.02], 0.64 |
| **Baseline EASI** | **1.07 [1.00–1.15], 0.039*** | 0.99 [0.94–1.05], 0.74 |
| **Baseline IgE** | 0.99996 [0.99992–1.00000], 0.06 | **1.00004 [1.00001–1.00009], 0.012*** |
| **TARC Improvement Rate** | **1.01 [1.00–1.02], 0.008*** | **0.99 [0.98–1.00], 0.0013*** |
| **IgE Improvement Rate** | **1.00 [1.00–1.02], 0.037*** | 0.997 [0.988–1.001], 0.16 |

(Model summary)

**EASI75 model**: Pseudo R² = 0.226, Model p = 0.0027, LOF p = 0.448.

**Adverse Event model**: Pseudo R² = 0.165, Model p = 0.0099, LOF p = 0.074

Notes:Multivariable logistic regression models were constructed separately for each outcome using covariates optimized for statistical significance and model fit.

*Statistically significant at p < 0.05.

# Supplementary Table S2

a) Variables Correlated with 3-Month PP-NRS Improvement in Patients Treated with JAK Inhibitors

| **TOP3 Variables Correlated with　 PP-NRS Improvement** | **Standardized β** | **Raw β [95% CI]** | ***p-*value** | **VIF** |
| --- | --- | --- | --- | --- |
| **EASI Improvement Rate** | **0.662** | **0.588 [0.288, 0.887]** | **0.0004*** | **1.75** |
| **IgE Improvement Rate** | –0.217 | –0.030 [–0.070, 0.009] | 0.1295 | 1.24 |
| **Eosinophil Improvement Rate** | 0.119 | 0.068 [–0.117, 0.253] | 0.4609 | 1.64 |
| Model Summary: N = 37, R² = 0.533 (adjusted R² = 0.439), RMSE = 20.33, model (p) = 0.0005, no multicollinearity (VIF < 2 for all variables) *Statistically significant (p < 0.05). | | | | |
| Notes: The full model included baseline PP-NRS, baseline IgE, and TARC improvement rate.  CI, confidence interval; PP-NRS, Peak Pruritus Numerical Rating Scale; IgE, immunoglobulin E; TARC, thymus and activation-regulated chemokine; VIF, variance inflation factor; RMSE, root mean square error | | | | |

a) Variables Correlated with 3-Month PP-NRS Improvement in Patients Treated with Anti-IL-13 Antibodies

| **TOP3 Variables Correlated with　 PP-NRS Improvement** | **Standardized β** | **Raw β [95% CI]** | ***p*-value** | **VIF** |
| --- | --- | --- | --- | --- |
| **Baseline PP-NRS** | **0.509** | **11.388 [6.220, 16.555]** | **<0.0001*** | **1.19** |
| **Baseline IgE** | **–0.421** | **–0.002 [–0.003, –0.001]** | **0.0015*** | **1.35** |
| **EASI Improvement Rate** | 0.254 | 0.602 [–0.118, 1.322] | 0.0981 | 2.06 |
| Model Summary: N = 38, R² = 0.665 (adjusted R² =0.600), RMSE = 45.28, model (p) = < 0.0001, no multicollinearity (VIF < 2 for all variables) | | | | |
| Notes: The full model also included Eosinophil improvement rate, IgE improvement rate and TARC improvement rate.  CI, confidence interval; PP-NRS, Peak Pruritus Numerical Rating Scale; IgE, immunoglobulin E; TARC, thymus and activation-regulated chemokine; VIF, variance inflation factor; RMSE, root mean square error | | | | |
